# Loyal Wingmen, Artificial Intelligence, and Cognitive Enhancement: A Warning against Cyborg-Drone Warfare

**DOI:** 10.1080/15027570.2025.2507458

**Published:** 2025-05-26

**Authors:** Christian Enemark

**Affiliations:** Department of Politics and International Relations, University of Southampton, Southampton, UK

**Keywords:** Artificial intelligence, brain-computer interface, cognitive enhancement, cyborg, drones

## Abstract

Some states are planning to acquire armed drones that incorporate artificial intelligence (AI) and fly alongside inhabited aircraft. The use of drones according to this “Loyal Wingman” concept is an example of tactical human-machine teaming, and it could be militarily advantageous in future aerial warfare. Incorporating AI into the operation of a weapon system’s critical functions (selecting and engaging targets) nevertheless carries an ethical risk: that a human will be unable to exercise adequate control over the use of force and unable to take responsibility for any injustice caused. To reduce this risk, one potential approach is to pursue “meaningful human control” over armed and AI-enabled drones by increasing their human supervisors’ cognitive capacity. The use of brain-computer interfaces (BCIs) to achieve such an increase might be beneficial from the perspective of military ethics if it enabled faster human interventions to prevent unjust, AI-associated harms. However, as this article shows, that benefit would be outweighed by the ethical downsides of waging cyborg-drone warfare: the undermining of pilots’ *hors de combat* noncombatant status and of human moral agency in the use of force.

## Introduction

In the conduct of aerial warfare, should airborne pilots use cognitive enhancement technology to supervise the functioning of armed and AI-enabled drones? This ethical question arises at the potential intersection of two technological trends in contemporary military affairs: the incorporation of AI into weapon systems, and the augmentation of human warfighters’ brainpower. One product of the first trend is the concept of “Loyal Wingman” drones. These aircraft, as elements of a “human-machine team,” would accompany inhabited aircraft into militarily contested airspace. Here, though, an ethical concern would be to ensure that AI involvement in the operation of the drones’ weaponry remained under adequate human control. Such control is required as a matter of military utility. And it is required also to preserve the potential in war for moral agency to be exercised and for moral responsibility to be borne for any injustices caused by violent action. But as AI technology becomes increasingly capable of rapidly performing functions within a weapon system, the risk is growing that human function-performers within that system will fail to keep up cognitively with the flow of AI-generated outputs. One idea for addressing that risk is to augment human cognition in a way that makes human-AI interaction faster and thus affords an adequate degree of human control over AI-assisted violence.

When it comes to the incorporation of AI technology into weapon systems, no government in the world has publicly advocated the complete delegation of system functions from humans to AI. Rather, some governments appear to be primarily interested in using AI technology to complement rather than replace human warfighters. The declared policy of the US and UK governments, for example, is to adopt AI for the purpose of “human-machine teaming” (Development, Concepts and Doctrine Centre 2018, v; Office of the Assistant Secretary of Defense for Acquisition 2018, 29). According to this concept, artificial (non-human) intelligence is regarded as being different from but combinable with “human cognition” (Ministry of Defence 2022a, 7). However, even in circumstances where humans and AI were collaborating as “teammates” in war, the deployment of an AI-enabled weapon system could still be considered morally unacceptable. This would be the case, as some scholars have argued, if the system functioned in the absence of “meaningful human control” (Amoroso and Tamburrini 2020; Moyes 2016). Non-meaningful (inadequate) control by humans would problematise the exercising of moral agency and the bearing of moral responsibility (Heyns 2016, 370; Leveringhaus 2016, 172).

An insufficiently controllable weapon system is an unwelcome prospect from a purely military perspective, too. Such a system, in which AI-generated outputs are highly unpredictable and excessively fast-flowing, would be difficult for the deploying state’s warfighters to trust, and it could also endanger those personnel. As a solution, some militaries have recently been considering the augmenting of humans’ capacity to keep up with AI information-processing (Kania 2019; Rice and Selman 2022). For example, a report on “Human/Machine Fusion” published in 2019 by the US Army’s Combat Capabilities Development Command provided long-term predictions about the military implications of physically integrating machines into the human body to enhance human performance (Emanuel et al. 2019). By 2050 or sooner, the authors claimed, emergent “cyborg” technology would include “neural implants for brain–computer interfacing” that enable “seamless interaction” between humans and weapon systems such as “drones” (Emanuel et al. 2019, 7). Two years later, the UK defence ministry’s Development, Concepts and Doctrine Centre and the German defence ministry’s Bundeswehr Office for Defence Planning jointly published a similar paper. This predicted that, within 30 years, “increasing use of autonomous and unmanned systems … could significantly increase the combat effect that an individual can bring to bear” (Development, Concepts and Doctrine Centre and Bundeswehr Office for Defence Planning 2021, 15). The realisation of this increase was predicted to depend critically upon enhancing “the interfaces between people and machines,” with “human augmentation” likely to “play an important part in enabling this” (Development, Concepts and Doctrine Centre and Bundeswehr Office for Defence Planning 2021, 15).

At present, such predictions are long-range and speculative. However, as some governments continue to pursue research on augmenting warfighters’ cognition, it is important to consider now what kinds of augmentations might be technically feasible, and which kinds (if any) could be ethically justified. In this article, attention focuses on the augmenting of human cognition by technological means. Ethical assessments thereof usually inquire into whether or how experimental research on cognitive enhancement should be undertaken, and moral concerns have been identified mainly from the perspective of human research ethics (Erden and Brey 2022). For example, where such research is undertaken for military purposes and involves military personnel, one potential problem is that a human subject of experimentation might not be free to provide or withdraw their informed consent (Latheef and Henschke 2020). As a matter of human research ethics, then, some kinds of cognitive enhancement might never be permitted to be developed to the point of being deployable during a war. However, this possibility need not preclude consideration, from the perspective of *military* ethics, of whether certain technologies for enhancing cognition should (*if* developed) be used.

This article advances the ethical debates about AI and human enhancement in warfare by focusing on one example of tactical human-machine teaming, the use of armed and AI-enabled drones as Loyal Wingmen, and by assessing the hypothetical idea of using brain-computer interfaces (BCIs) to conduct cyborg-drone warfare. The first part of the article introduces the Loyal Wingman concept and outlines the potential advantage of having a nearby human supervising a drone rather than having a faraway human controlling a drone. This is followed by a discussion of the ethical risk that Loyal Wingmen might operate in the absence of meaningful human control, and of whether this risk could be reduced by augmenting human cognition. After consideration of the potential for BCIs to bind human-machine teams and enable cyborg supervision of AI, the discussion concludes with a warning about the potential for “cyborg-drone warfare” to generate two ethical problems: the indiscriminate harming of a pilot who is captured, and the diminishment of a cyborg’s moral agency.

## The turn to human-machine teaming in drone warfare

During the last two decades, the use of armed drones in war has mostly involved air-to-ground missile strikes in Afghanistan and Iraq. Here, drone-using states (mainly the United States and the United Kingdom) have not had to contest the local airspace, so remote-controlled aircraft like the MQ-1 Predator and MQ-9 Reaper have been able to operate from a position of unchallenged dominance. In less permissive environments, and when confronting more powerful adversaries, drone warfare of this kind would be much harder to sustain. The long-range communication links between in-theatre drones and their distant operators are not conducive to aerial combat success, and drones like the Reaper would be more vulnerable to the enemy’s air defence systems. Moreover, for US and European air forces, it might not be the case in future that airspace domination can be won and maintained by instead using inhabited aircraft. As the size of national air fleets are reduced to cut costs, a strategic concern is growing that too few fighter jets would be available for use in a conflict against a powerful military like China’s (Jordan 2021, 8; Penney 2022).

A desire to prevail against Chinese defensive and offensive capabilities is what partly motivates the US military’s Third Offset strategy, which focuses on “employing machines [AI] to aid human decision making and execute military operations” (Schmidt and Work 2022). This focus is reflected in a 2018 Defense Department prediction that “[m]ilitary operations of the future will require collaboration between unmanned systems and humans” (Office of the Assistant Secretary of Defense for Acquisition 2018, 29). Similarly, the UK Ministry of Defence has anticipated that “future military advantage” will depend upon “the effective integration of humans and machines into war fighting systems that outperform our opponents” (Development, Concepts and Doctrine Centre 2018, v). It appears to be the case, then, that the US and UK governments do not currently envisage humans being *entirely* replaced by AI. Rather, as reflected in Paul Scharre’s idea of “centaur warfighting” (Scharre 2016), these governments’ strategic objective is apparently to arrange a complementary relationship: AI data-processing combined with human judgment-making.

Human-machine teaming is exemplified, at the tactical level, by the idea of AI-enabled drones “collaborating” with the airborne pilots of military aircraft. It is an idea which, if realised, carries the potential to ensure that sufficient mass for operations will be available even once fewer human pilots are taking to the air. States that use drones as Loyal Wingmen would then, it is hoped, be better able to withstand aircraft losses and prevail “in a peer-level conflict” (Penney 2022). The Loyal Wingman concept is central to the forthcoming “sixth generation” of fighter jet systems being developed by several air forces. These include the Tempest system (United Kingdom, Italy and Japan) (Beale 2022), the Next Generation Air Dominance programme (United States) (Jordan 2021, 1), and the Future Air Combat System (France, Germany and Spain) (Stevenson 2019). A core assumption underlying the development of all such systems is that inhabited combat aircraft will operate in coordination with various drones that provide immediate support and protection. This could be in the form of reconnaissance, electronic warfare, kinetic firepower, or (at minimum) by acting as decoys (Losey and Demarest 2023). One of the most advanced versions of a Loyal Wingman today is the Kratos XQ-58A Valkyrie, which has been developed for the US Air Force (USAF) to fly alongside existing (fifth generation) fighters like the F-22 and F-35 (Venckunas 2022). The Valkyrie drone can reportedly carry “two GBU-39 small-diameter bombs” for air-to-ground strikes (Saballa 2023), and many of its functions are controllable by an AI system called “Skyborg” (Axe 2021).

In the aerial human-machine teams envisaged by former USAF Secretary Frank Kendall, up to five drones would fly alongside one US fighter jet (Hadley 2022). The human in the jet would not, however, manoeuvre the Loyal Wingmen or operate their sensors on an ongoing basis. Rather, that pilot would contribute to the team by “managing” the drones (Penney 2022); a role which Kendall has compared to that of a “quarterback” who calls plays in a game of American football (Schake 2023). At the outset, for example, the pilot might issue standing instructions for their Loyal Wingmen to fly in a particular direction and search for certain kinds of objects (potential targets). From a communication perspective, situating a human supervisor nearby – who could, if necessary, seize control of a drone’s functions from its AI – would be operationally superior to relying upon a remotely located operator. This is because data transmission between transceivers over shorter distances is harder for an enemy to disrupt with jamming signals (Warrington 2022).

In addition, as between an airborne pilot and their Loyal Wingman, data from the drone’s sensors (such as video images) and control signals from the fighter jet would transmit slightly faster than is the case when data must flow via satellite between continents. Although the difference in speed is only a few seconds (Cockburn 2016, 5), the time delay associated with long-range drone warfare makes it harder for drone operators to hit a fast-moving target. That latency also carries the risk of causing unintended harms. That is, if a civilian suddenly enters the target zone of a launched missile, there are fewer seconds available to abort or divert the attack. By comparison, the physical proximity of a Loyal Wingman would be morally advantageous to the extent that it enabled humane interventions to be timelier than is the case when an armed drone and its human operator are very far apart.

## Loyal Wingmen and the problem of control

From an ethical perspective, the more significant aspect of the Loyal Wingman concept is the incorporation and influence of AI technology. The central concern for the future is that armed and AI-enabled drones, despite putatively “teaming” with a nearby human, might not actually operate under conditions of adequate human control. Maintaining such conditions is important for humanitarian reasons and because, as Heather Penney (2022) has insisted, “[h]umans [in fighter jets] will need confidence that their [drone] teammates will consistently … defer to their human’s control – just as a [human] wingman defers to their lead in combat.”

Before a military operation occurs, there are some limitations that can be imposed on an AI-enabled weapon system. Pre-programmed restrictions could include, for example: measures to limit the duration of time and/or the geographical range within which a platform can operate; and measures to control the types of targets that a system can identify and engage (ICRC 2021). But beyond that, once combat is underway, human control becomes largely a matter of tactical decision-making. AI is inherently incapable of making moral decisions and bearing moral responsibility, and it cannot replicate a human’s ability to exercise judgment (based partly on lived experience). Therefore, the degree of human control over the AI-enabled and violence-related functions of a weapon system needs to be meaningful enough, under the circumstances, to preserve the possibility of morally responsible use.

This requirement is, to some extent, already reflected in the policies of two states that plan to deploy Loyal Wingmen: the United States and the United Kingdom. In the terms of a 2023 US Defense Department Directive on Autonomy in Weapons System, armed drones of this kind appear to fall within the category of “operator-supervised autonomous weapon systems” (Hicks 2023, 22). Such systems are “designed to provide operators with the ability to intervene and terminate engagements, including in the event of a weapon system failure, before unacceptable levels of damage occur” (Hicks 2023, 22). Thus, according to this policy, a US Loyal Wingman would need to be designed “to allow commanders and operators to exercise appropriate levels of human judgment over the use of force” (Hicks 2023, 3). Similarly, the UK Ministry of Defence policy is to “ensure that delegation of tasks or decisions to AI systems does not lead to a “responsibility gap” between systems that take decisions or make recommendations, and the human commanders responsible for them” (Ministry of Defence 2022b, 4). To avoid this gap, “there must be context-appropriate human involvement in weapons which identify, select and attack targets” (Ministry of Defence 2022b, 3), and the UK’s policy also describes “the ability to exercise human judgement” over such systems’ outcomes as “essential” (Ministry of Defence 2022b, 10).

In both states’ policies, the word “appropriate” implicitly rejects the notion that *any* form or degree of human involvement in a weapon system’s operation is necessarily sufficient. And that word also conveys an understanding that the circumstances in which a weapon is deployed are morally relevant. Human involvement of a kind that is appropriate in one situation might be inappropriate (i.e. either inadequate or excessive) in another situation. For present purposes, then, it is worthwhile taking the Loyal Wingman concept as an example and considering whether, in the future, the technological augmentation of human cognition would be a morally permissible circumstance for the control of armed drones. Would this arrangement satisfy the principle that AI-enabled weapon systems should be under meaningful human control?

The overall operation of a Loyal Wingman would include various benign functions (take-off and landing, navigation, and in-flight manoeuvring), and these could be performed by AI technologies without raising any serious ethical concerns. But a more worrying scenario was envisaged by Jon Norman, a vice president at Raytheon Technologies, in a speech at the 2022 Farnborough Air Show in the United Kingdom. He reportedly said that, in future battles, AI might allow a pilot to send armed drones close to an enemy position “and have them just fire at will” (Chan 2022). Provided that a drone’s critical functions (selecting targets and engaging targets) could *only* be performed *with* human involvement, such a weapon system could begin to be regarded as being under meaningful human control. In addition, though, the meaningfulness of human control over AI-assisted violence needs to be understood in a temporal sense: an opportunity must exist to override AI *in time* to prevent unintended effects and unjust harms resulting from system errors. The condition of meaningful human control might thus involve, for example, having time for a human to check whether the AI performer of an intelligence function has correctly tagged a target as “legitimate.” Or, it might involve preserving the opportunity for careful checking of an AI-generated estimate of the damage likely to be caused if the weapon system were used under specific circumstances.

It might the case in practice, however, that there is insufficient time to take such care. A problem with the Loyal Wingman concept, according to one analysis, is that “it’s not clear that fighter pilots in action have time to manage semi-autonomous helpers” (Perrett 2021). Indeed, in a combat environment full of distractions, if a drone’s AI rapidly provided a list of targets for its pilot-supervisor to choose from, the illegitimacy of one listed target (for example, a civilian aircraft wrongly tagged as an enemy threat) might be overlooked in the rush. Already, there exist AI-enabled weapon systems which operate so quickly that their human controllers feel outpaced and overwhelmed (Bode and Watts 2021). And, in high-pressure situations of human-machine teaming in the future, a lack of opportunity for human deliberation and timely intervention could exacerbate another factor which undermines the adequacy of human control: over-trusting AI.

Research in the field of Human–Computer Interaction has long recognised the modern-day human tendency towards “automation bias” (Skitka, Mosier, and Burdick 1999). This bias can lead people to overestimate the reliability of an AI-generated output and to devalue or ignore contradictory information. Sometimes, excessive trust in AI might simply arise from human laziness and complacency, and otherwise it can be attributable to unfavourable circumstances. If humans become confused by fast-moving and complicated situations, they might feel that they have no choice but to trust an AI technology that might not actually be *worthy* of trust. In which case, the meaningfulness of human control and the purchase of moral agency are undermined, because the bestowing of “trust” in AI is forced and therefore false. This form of over-trusting was probably the cause, for example, of some past incidents of fratricide involving the Patriot air defence system, where human operators failed to override the system’s false identification of friendly aircraft as enemy targets (Burt 2018, 50; Piller 2003).

As warfare becomes more heavily data-driven, warfighters can also experience information overload. This, too, has the potential to induce excessive trust in the AI-generated outputs of military systems, because human operators might be tempted to “use AI tools as a means to offload cognitively” (Johnson 2023, 52). In the case of AI-enabled Loyal Wingmen, and as acknowledged by the director of Boeing’s Airpower Teaming System programme, the transferring of data from a drone to its human supervisor’s aircraft might cause information overload (Insinna 2020). If that were to happen, and AI thus became over-trusted, it would make human control less meaningful and undermine the supervising pilot’s capacity to be morally responsible for the use of force. A capacity for moral responsibility, including the ability to be held accountable for wrongdoing, is an important element of what it means to be genuinely in control of a weapon system.

The potential for meaningful human control is partly determined also by the way in which a weapon system is designed to account for so-called “human factors.” For the US and UK governments, preparation to deploy human-machine teams effectively is regarded, firstly, as a matter of providing the necessary training to warfighters. In February 2023, for example, a US State Department declaration on responsible military use of AI included the statement that “personnel who use … military AI capabilities” should be “trained so they sufficiently understand the capabilities … and can make context-informed judgments on their use” (Bureau of Arms Control, Verification and Compliance 2023). Similarly, among the UK government’s Ethical Principles for AI in Defence, the “Understanding” principle requires that “AI-enabled systems, and their outputs, must be appropriately understood by relevant individuals” who “must be suitably trained and competent” (Ministry of Defence 2022b, 10). Secondly, both governments have recognised that the effectiveness of trained warfighters depends also upon the design of human-AI interface mechanisms within a weapon system. According to the UK Ministry of Defence, “good human-machine teaming will require intuitive human-machine interfaces” (Development, Concepts and Doctrine Centre 2018, 32). And it is US policy to use a “human-machine interface” that is “readily understandable” so that system operators can “make informed and appropriate decisions regarding the engagement of targets” (Hicks 2023, 4). Indeed, if interfaces between the human and AI elements of a system made the latter more understandable to the former, there would seem to be greater scope for maintaining meaningful human control.

In the case of human-machine teaming involving Loyal Wingmen, one suggestion is that it would not be sufficient in a contested airspace for the supervising pilot to “just strap an iPad onto [their] leg and go from there” (Losey 2022). Rather, that pilot would need “fully integrated and elegant interfaces to be able to supervise, direct and control” their nearby drones (Losey 2022). Such interfaces might initially include familiar cockpit features like buttons, dashboards and touchscreens for data input, and perhaps a head-up display inside a flight helmet to project incoming data. In the future, though, if the AI technology within Loyal Wingman systems becomes more advanced and their operating environments become more challenging, the required mechanisms of human-AI interfacing could become more complex (Puscas 2022, 3). Then, at some point, human control over multiple drones might no longer be sustainable in a meaningful way, even when attempted by a highly trained pilot who can interact quickly with AI via a highly intuitive interface. The pace and complexity of close-control drone warfare will have exceeded the natural limit of humans’ cognitive capacity.

The human brain is limited in its ability to process information, and a human’s cognitive performance declines over time while they focus attention on tasks and make decisions (Herlihy 2022, 79–80). For these reasons, some states that plan to incorporate more and better AI technology into their weapon systems are concerned to achieve what the UK Ministry of Defence has called “manageable cognitive loads” for system operators (Development, Concepts and Doctrine Centre 2018, 15). The increasingly fast-paced and data-driven character of modern warfare presents a serious challenge in this regard. And, from an ethical perspective, there is a danger that the cognitive task of making moral decisions based on morally significant information will more often be an unmanageable one for human warfighters. The neurotechnology already exists to measure and monitor a person’s cognitive load, and one suggested possibility for human-machine teaming is that a machine could be programmed to “take on work where [human] operators become overloaded” (Development, Concepts and Doctrine Centre 2018, 32). This is reportedly a design feature of the Tempest fighter jet being developed for the Royal Air Force (RAF). Sensors placed inside the pilot’s helmet would monitor brain signals and, if these indicated that the pilot was becoming overwhelmed by stress or had lost consciousness, “the AI could take over” (Dempsey 2022).

Currently, the potential for a human to be cognitively overwhelmed, during combat, by the need for high-speed reactions to events is partly a function of interface design. Compared to AI, human controllers can seem slow when their interaction with a system can only occur through bodily movement: for example, by using hands, fingers or mouth to flick a switch, touch a screen or speak an instruction. A radical alternative, which appears to be garnering research interest within some militaries, is to bypass these traditional mechanisms of human-machine interfacing. By instead using brain-computer interface (BCI) devices – located on or inside a warfighter’s head – data flows might be made more efficient, and the degree of human control over AI-enabled systems might be increased. If this idea were ever realised, though, there could still be some serious ethical risks associated with the deployment of cyborg pilots alongside Loyal Wingman drones.

## Brain-computer interfaces for cyborg-drone warfare

Facing the prospect of data-rich and AI-assisted warfare being conducted at super-human speed, one possibility is that military planners will eventually feel forced to choose between human control and AI control of advanced weapon systems (Saxon 2016, 206). Against this, it has been suggested that meaningful human control could in future be maintained by imposing “built-in limitations” on AI (Gillespie 2020, 62). Designing a system which limits the speed of AI data-processing, for example, could reduce the likelihood of human controllers becoming cognitively overwhelmed. However, both visions of the future are problematic. On the one hand, abandoning human control could sometimes be militarily too risky and ethically unacceptable. On the other hand, commitments to engineer the retardation of AI could clash with operational imperatives to outpace an enemy’s decisions and actions during combat. Therefore, it is worth considering a third approach to pursuing meaningful human control of AI-enabled weapon systems: improving (rather than replacing or accommodating) human cognition. BCIs are among the most technologically ambitious mechanisms for potentially achieving such improvement. And such devices are arguably most likely to be used first (if ever) by those warfighters who traditionally interact most intensively with computerised systems: air force pilots.

According to the tactical logic of human-machine teaming, the military advantage of using fighter jets alongside Loyal Wingmen lies in the combining of their respective strengths. The idea is that, by “partnering” with drones, human pilots would be freed to “focus on critical cognitive tasks, such as dealing with unforeseen events and managing battlespace operations” (Penney 2022). However, it still might be difficult sometimes to keep on performing those critical tasks successfully. Even without the added complication of monitoring as many as five Loyal Wingmen, a combat pilot’s cognitive performance can become degraded by the requirement to remain highly vigilant for long periods while waiting for an attack or an opportunity to act (Goodley 2020, 19).

To mitigate this risk, military researchers have found that getting plenty of sleep and staying well-nourished can help an individual to improve and sustain their situation awareness, threat detection and decision making (Brunyé et al. 2020, 453). Yet these practices do not count as “cognitive enhancement” in the sense of being “the amplification or extension of core capacities of the mind” (Bostrom and Sandberg 2006, 2). By contrast, for many decades there has been a persistent practice of using pharmacological interventions to *temporarily* enhance pilots’ attention and alertness. During World War Two, for example, the RAF issued Benzedrine (an amphetamine) to British aircrews to keep them awake during lengthy missions (Pugh 2018). And the USAF authorised pilots’ use of dextroamphetamine to counter fatigue during the Vietnam War and the 1991 Gulf War (Kenagy et al. 2004; Working Group of the Royal Society 2012, 34). More recently, in response to concerns that amphetamine use might cause impaired judgment and drug addiction, the USAF stopped authorising it and opted instead for the alertness drug Modafinil (Burkeman and Norton-Taylor 2003; Evans 2021, 41).

In the emergent era of human-machine teaming, one problem with relying upon pharmacological technology to enhance cognition is that a drug (ingested or injected into the body) takes time to influence the brain. Thus, drug-based enhancement is generally unsuitable for use at extremely short notice. Moreover, the ability of a pilot to adequately supervise multiple Loyal Wingmen would likely depend upon much more than sustained alertness. Beyond that, the maintenance of meaningful human control could require cognition-enhancing technology that is designed to enable the rapid flow of data between humans and machines.

An electronic BCI device enables two-way communication between a human brain and a machine. It reads a person’s neural signals and converts them into computer-readable code, and it converts code into signals that activate certain parts of the brain (Evans 2021, 42). As such, using a BCI continues a long tradition of “cyborgisation” whereby non-biological resources are melded with the human body and mind (Clark 2003). Brain signal acquisition can be accomplished invasively by surgically implanting a device in the human cortex, or non-invasively by connecting one to a person’s scalp. The latter method, though, is currently less effective because the skull tends to reduce the strength of electrical signals (Binnendijk, Marler, and Bartels 2020, 8). Either way, because the input and output of data-bearing signals depends on brain activity rather than muscle movements, BCI technology can eliminate the need for physical control mechanisms like keyboards and joysticks. Thus, human-machine interaction is enabled to occur in new and faster ways. Implanted neural devices have already been used experimentally by injured people to control prosthetic limbs (Ellery 2023; Gallagher 2019). And, when combined with AI technology, BCIs hold the potential for human users to access and communicate huge quantities of information. Such a form of cognitive enhancement is predicted to be useful, for example, in reducing the time and number of lawyers required to work on complex legal cases (Ames 2022).

In addition, and for similar reasons, there is continuing interest in the militarisation of BCIs as a technology for improving human-machine teaming (Development, Concepts and Doctrine Centre and Bundeswehr Office for Defence Planning 2021, 91; Kosal and Putney 2023). The idea of “mind-controlled” drones has been around for over a decade (Axe 2019). A report published in 2012 by the UK-based Royal Society found that the potential military applications of “brain–machine interfaces” included using them to “operate robots or unmanned vehicles in hostile territory” (Working Group of the Royal Society 2012, 39). The US Army’s *Cyborg Soldier 2050* report of 2019 speculated that “in the relatively near future, the U.S. military will use brain–machine interfaces that will enable soldiers to directly control assets, such as drones” (Emanuel et al. 2019, 7). And a study published by RAND in 2020 predicted that, on the “future battlefield,” human thoughts could be “channelled to AI software” via BCI capabilities, allowing operators to “control multiple technological platforms simultaneously” (Binnendijk, Marler, and Bartels 2020, 3). Although it is difficult to be confident that such predictions will be realised soon or ever, it is still worthwhile to start considering now whether a cyborg approach to the control of AI-enabled weapons is ethically desirable. Such consideration is relevant to the question of whether cyborgisation for human-machine teaming is something that governments should be researching at all. Accordingly, the final section of this article addresses the question: if a fighter pilot used a BCI to supervise several Loyal Wingmen, what might be the relative benefit and risk from the perspective of military ethics? The answer, arguably, is that cyborg-drone warfare would on balance be morally disadvantageous and ought therefore to be avoided in the future.

## A warning

Cognitive enhancement can also enhance the morality of war (from a *jus in bello* perspective) if it facilitates greater distinction between combatants (why may legitimately be targeted) and civilians (who may not). When it comes to air-to-ground strikes by an AI-enabled drone, it is already more beneficial to civilians near a strike zone to have a pilot-supervisor positioned close to the drone rather than far away from it. By avoiding the signal latency associated with satellite-based communication, data can flow more quickly between weapon system and human controller, thus allowing more time for intervention if this is needed to spare civilians. Adding to this humanitarian benefit of drone warfare conducted in Loyal Wingman mode, the use of a BCI could hypothetically accelerate the pilot’s decision and action to intervene. This is because humans’ neural (in-brain) reactions are typically faster than their kinetic (bodily) reactions (Evans 2021, 44; Kosal and Putney 2023, 87). For the same reason, during air-to-air combat, a drone’s cyborg supervisor could more quickly override the initiation of a missile strike if a civilian aircraft were falsely identified (by AI) as a threat.

Weighing against this potential benefit, cyborg-drone warfare could also usher in at least two serious ethical risks. The first risk, associated only with BCIs which are surgically implanted, is that a pilot who is captured might suffer indiscriminate harm. While it is generally permissible for enemy forces to intend harm against combatants, a person ceases to be a legitimate target when they are rendered *hors de combat*. This change can ordinarily occur when a combatant is captured, disarmed, and imprisoned. However, in the absence of a sufficiently strong jamming signal, an interface device that has become part of that pilot’s body could provide constant connectivity with (and control over) a nearby Loyal Wingman and its onboard weapons. In that case, an enemy might reasonably regard a known and unjammable cyborg as posing a continuing military threat, even while in captivity (McAllister 2019, 72). Non-combatant status would not have been acquired at the moment of capture, and the enemy would retain moral permission to neutralise the pilot’s brain as a source of signals that could summon a drone strike. This might be done simply by killing the pilot quickly, or (if the enemy were willing to risk taking more time) by conducting a coerced surgical procedure to remove the implanted BCI. From the perspective of *in bello* ethics, the problem in this scenario is that the legal protections traditionally afforded to a prisoner of war (a category of non-combatant) could be undermined. In this hypothetical future, that is, captured cyborg pilots might be denied non-combatant status even if they have and express no intention of fighting on. And, more generally, an enemy’s fear of human-AI interfacing could prompt the swift and indiscriminate harming of *all* captured pilots (cyborgs and non-cyborgs alike) on the mere suspicion of their harbouring a BCI which connects them to a weapon.

The second ethical risk associated with using BCIs to supervise Loyal Wingmen is that these devices (whether implanted or not) might diminish a pilot’s moral agency during combat. The risk of diminished agency has previously been highlighted in relation to pharmacological approaches to cognitive enhancement. Jessica Wolfendale has argued, for example, that administering certain performance-enhancing drugs to soldiers might also produce a mental condition of dissociation. A potential result of this might be the commission of atrocities by a perpetrator who is not fully aware of what they are doing (Wolfendale 2008, 35). This risk has been associated with the use of some amphetamines, especially (Holley 2015; Saniotis and Kumaratilake 2020, 69). By contrast, in the circumstance of BCI-based augmentation of the brain’s data-processing capacity, the diminishment of moral agency could instead result from a cyborg’s sensation of cognitive convergence with AI technology. In this way, through the experiential blurring of artificial and human intelligence, the use of BCIs might compound rather than alleviate the problem of humans’ excessive trust in AI. This, in turn, could diminish rather than preserve the adequacy of human control over an AI-enabled weapon system.

Some analysis has suggested, to the contrary, that meaningful human control will in future depend critically upon BCIs. For example, Nørgaard and Linden-Vørnle (2021, 103) have argued that “the responsibility gap can be bridged in cyborg and neurocognitive battle networks keeping the human operator “in the loop” applying legal and ethical judgment.” And Anika Binnendijk, Marler, and Bartels (2020, 13–14) have predicted that BCIs could become “the only way to have humans remain effectively engaged in decisionmaking in war and keep pace with machines.” However, in the context of a human-machine team comprised of two different kinds of intelligence, it would not be prudent to assume that humans will forever continue to be the *leading* thinkers. Another possibility is that those humans who are neurally connected to an AI-enabled weapon system could become, in essence, followers (rather than leaders) of AI. As Robert Sparrow and Adam Henschke have argued, the future “cyborg soldier” could become comparable to a minotaur (a mythical creature with a human body and a non-human head) in the sense of being critically dependent upon AI-generated directions during combat operations (Sparrow and Henschke 2023, 116).

Here, it is worth emphasising that the imagined BCI-based interaction between a fighter pilot and a Loyal Wingman would not only involve the sending of control signals from human to drone. It would also involve that drone’s AI sending data directly into the pilot’s brain. Human senses would thus be bypassed, and information would be received more quickly than if it first needed to be displayed upon and read from a screen. Yet, in this removal of the traditional layers of mediation between human thought and AI data-processing, the potential would increase for a cyborg to feel cognitively “at one” with the AI that they are putatively supervising. In this scenario, because of the human-machine intimacy afforded by BCI technology, the idea of “overriding” AI-generated outputs could then cease to make any sense in the human’s mind. At this moment of cognitive convergence, human moral agency would no longer have an *independent* influence over the AI-enabled weapon system. The Loyal Wingman would not be under meaningful human control.

## Conclusion

Strategic pressure to be ready for a major conflict is driving some states to develop a new concept of drone warfare. In the design and assembly of air forces for the future, Loyal Wingmen are emerging as vital platforms for adding mass and firepower in support of small numbers of inhabited jets fighting for aerial dominance. The critical issue, from an ethical perspective, is whether the AI-enabled drones within these human-machine teams would operate under meaningful human control. If they did not, there would be reason for concern that wartime injustices caused by the operation of Loyal Wingmen could occur with impunity due to a responsibility gap.

As technology advances and warfare becomes faster, there is a risk that human controllers of weapon systems will find it increasingly difficult to understand what a system’s AI is doing and to know when overriding it is morally necessary. In the future, perhaps, even highly trained personnel using highly intuitive human-AI interfaces will struggle to exercise meaningful control and be genuinely responsible for the use of force in wartime. At that point, the natural limits of human cognition will have been reached. But the morally preferable response to this possibility is not necessarily to plan for cyborg-style enhancement of human warfighters’ brains. A pilot’s use of BCI technology to supervise Loyal Wingmen might, hypothetically, afford an increased opportunity for rapid intervention to spare civilians from AI targeting errors. However, this humanitarian benefit would arguably not be justification enough for resorting to cyborgisation if that were also to introduce serious ethical risks. Cyborg-drone warfare would be unjust and should be avoided if the broader effect of BCIs was to undermine non-combatant status and human moral agency. A more prudent approach to the future use of AI-enabled drones is to continue requiring that they be meaningfully controllable without this kind of human augmentation.
